# Peraldehyde

**Published:** 1886-02-20

**Authors:** 


**Peraldehyde.**—(New England Medical Monthly) I wonder if the many readers of the Monthly appreciate fully the great value of the new hypnotic, peraldehyde? In my hands it has proved simply invaluable. It is the hypnotic *par excellence.* In doses ranging from twenty to eighty drops, we can get most delightful sleep without after effects. I have given it to little babes and the result was all that could be desired. My mode of administration has been simply mixed with a little sweet water. If pure, it tastes not unlike peppermint and not at all disagreeable. It warms the stomach, and gives a most delightful sense of rest. To the weary doctor it is a God-send.

